# Supplementary material for: Gut microbiome dynamics and predictive value in hospitalized COVID-19 patients: a comparative analysis of shallow and deep shotgun sequencing
Source: Front Microbiol. 2024 Jun 19;15:1342749. doi: 10.3389/fmicb.2024.1342749 (PMC11219902; doi:10.3389/fmicb.2024.1342749)
Supplement: Supplementary file 1 [file Data_Sheet_1.docx]

Supplementary Material

Gut Microbiome Dynamics and Predictive Value in Hospitalized COVID-19 Patients: A Comparative Analysis of Shallow and Deep Shotgun Sequencing

**Katarzyna Kopera^1^†, Tomasz Gromowski^1,2^†, Witold Wydmański^1,3^†, Karolina Skonieczna-Żydecka^4^, Agata Muszyńska^1^, Kinga Zielińska^1^, Anna Wierzbicka-Woś^5^, Mariusz Kaczmarczyk^5,6^, Roland Kadaj-Lipka^7^, Danuta Cembrowska-Lech ^4,5^, Kornelia Januszkiewicz^4^, Katarzyna Kotfis^8^, Wojciech Witkiewicz^9^, Magdalena Nalewajska^9^, Wiktoria Feret^10^, Wojciech Marlicz^5,11^, Igor Łoniewski^1,4,5^, Paweł P. Łabaj^1^, Grażyna Rydzewska^7^*, Tomasz Kościołek^1,12^***†These authors share first authorship

^1^Małopolska Centre of Biotechnology, Jagiellonian University, Kraków, Poland

^2^Department of General Biochemistry, Faculty of Biochemistry, Biophysics and Biotechnology, Jagiellonian University, Kraków, Poland

^3^Faculty of Mathematics and Computer Science, Jagiellonian University, Kraków, Poland

^4^ Department of Biochemical Science, Pomeranian Medical University, Szczecin, Poland

^5^Sanprobi Sp. z o.o. Sp. k, Poland

^6^Department of Clinical and Molecular Biochemistry, Pomeranian Medical University, Szczecin, Poland

^7^Department of Internal Medicine and Gastroenterology, Central Clinical Hospital of the Ministry of Interior and Administration, Warsaw, Poland

^8^Department of Anesthesiology, Intensive Care and Pain Management, Pomeranian Medical University, Szczecin, Poland

^9^Independent Public Regional Hospital, Szczecin, Poland

^10^Clinical Department of Nephrology, Transplantology and Internal Medicine, Pomeranian Medical University, Szczecin, Poland

^11^Department of Gastroenterology, Pomeranian Medical University, Szczecin, Poland

^12^Department of Data Science and Engineering, Silesian University of Technology, Gliwice, Poland

*** Correspondence:**Tomasz Kościółek
tomasz.kosciolek@uj.edu.pl
Grażyna Rydzewska
grazyna.rydzewska@cskmswia.pl

## Supplementary Figures


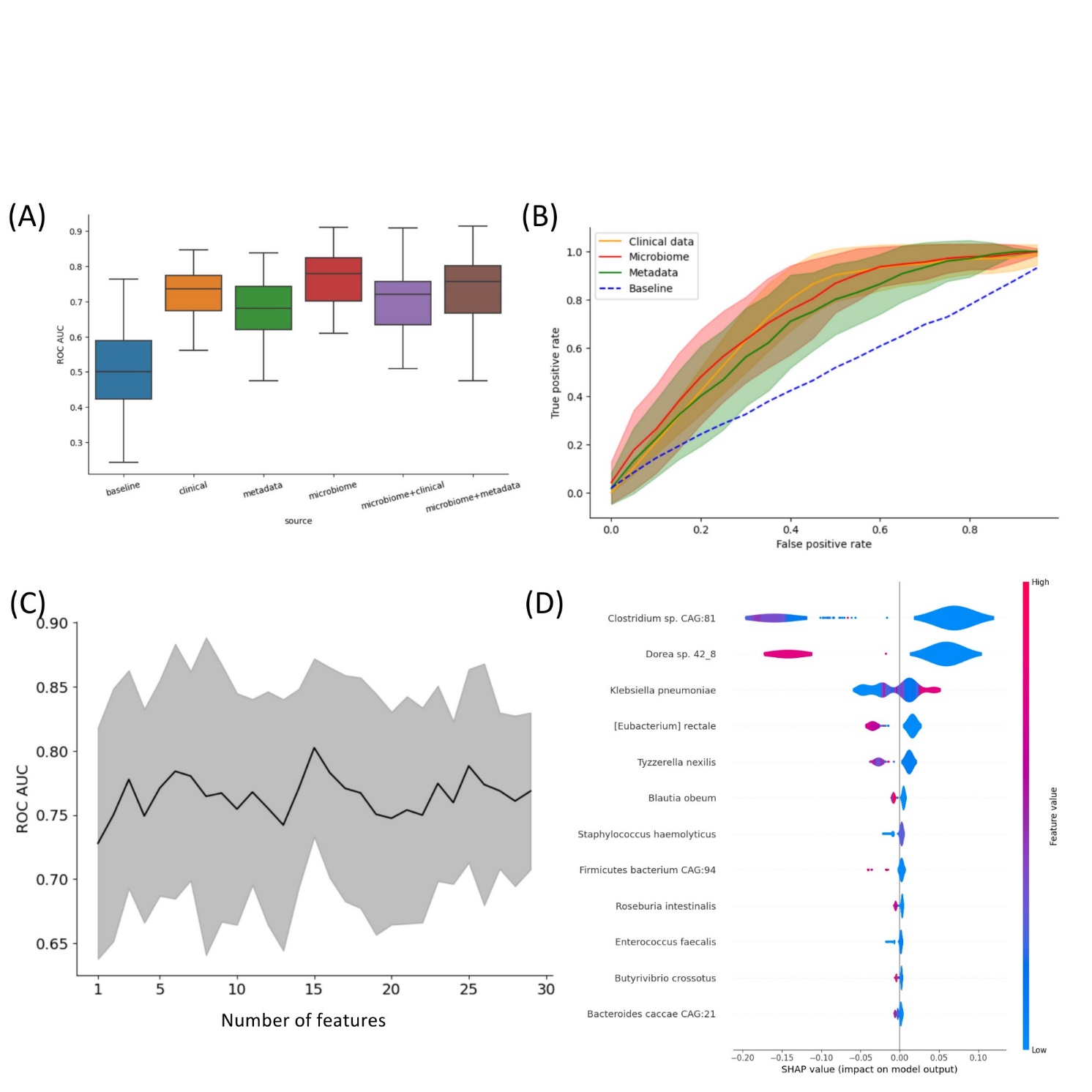


**Supplementary Figure 1. Insights into what influences the predictive power of patients’ outcomes (life vs death) classifier.** (A) Impact of different types of data on predictive power of the classifiers. This plot shows that access to microbiome data increases the performance of the classifiers. The three underlined classifiers form a cluster with no inter-cluster difference. (B) ROC curve of classifiers grouped by access to data. (C) Increasing number of metagenomical features doesn’t improve ROC-AUC beyond the first most important taxon. (D) Shapley values of the most important features for classification. Klebsiella pneumoniae is strongly connected to increased chance of patient’s death, as well as staphylococcus haemolyticus.


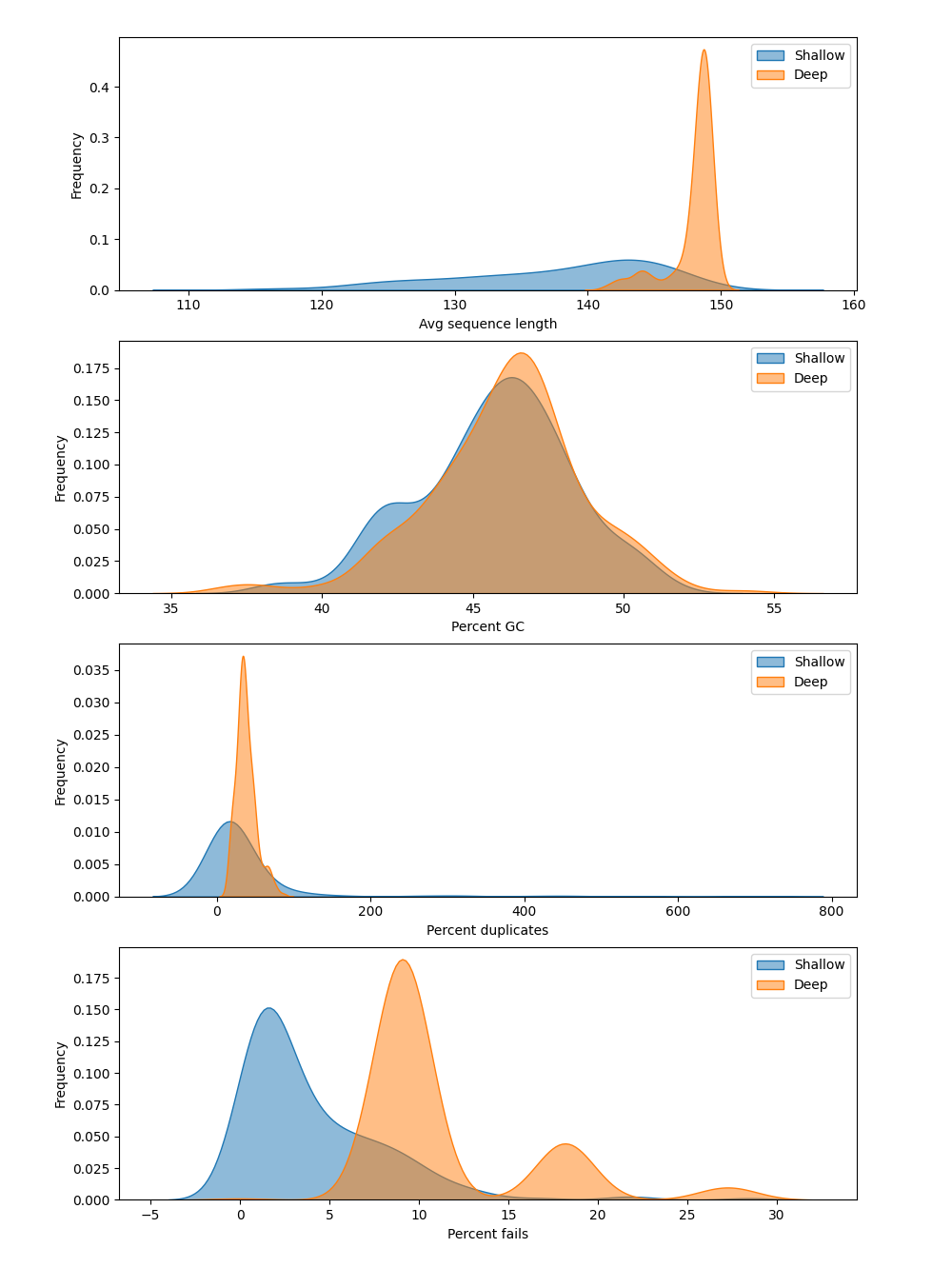


**Supplementary Figure 2.** **Quality comparison of matched shallow and deep sequencing samples.**


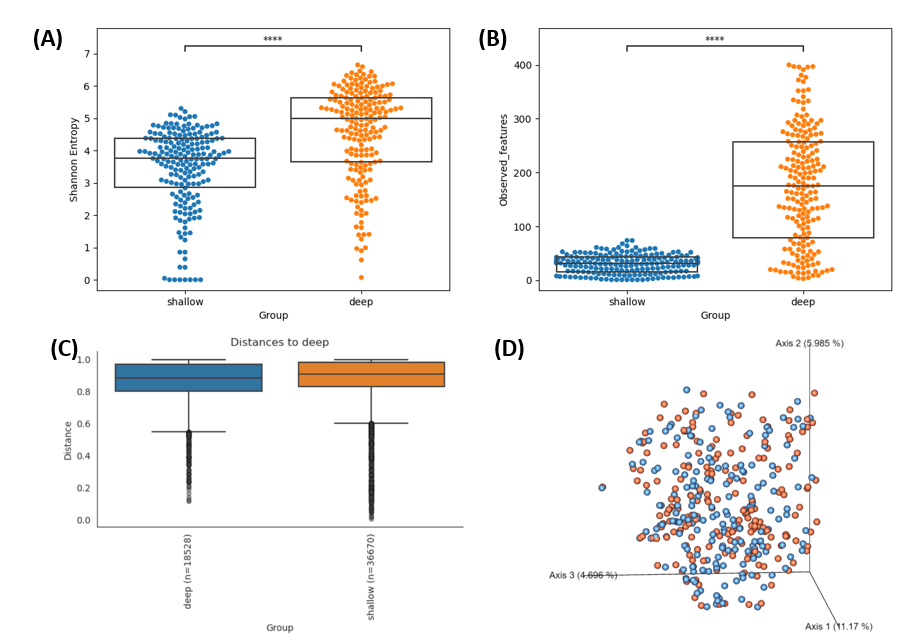


**Supplementary Figure 3. Comparison of shallow and deep sequencing samples in terms of a) alpha diversity (Shannon entropy) and b) number of observed features. c) Bray-Curtis beta diversity. d) Emperor plot of Bray-Curtis beta diversity.** Red: shallow, blue: deep sequencing.

1. **Supplementary tables**

| **Days elapsed from the day 0** | **Sample count** | **Percentage** | **Sample count after rarefaction** | **Percentage after rarefaction** |
| --- | --- | --- | --- | --- |
| 0 | 213 | 100.0 | 153 | 100.0 |
| 1 | 31 | 14.6 | 23 | 15.0 |
| 2 | 47 | 22.1 | 39 | 25.5 |
| 3 | 52 | 24.4 | 41 | 26.8 |
| 4 | 41 | 19.2 | 29 | 19.0 |
| 5 | 47 | 22.1 | 39 | 25.5 |
| 6 | 31 | 14.6 | 25 | 16.3 |
| 7 | 21 | 9.9 | 13 | 8.5 |
| 8 | 19 | 8.9 | 12 | 7.8 |
| 9 | 26 | 12.2 | 20 | 13.1 |
| 10 | 14 | 6.6 | 14 | 9.2 |
| 11 | 15 | 7.0 | 13 | 8.5 |
| 12 | 9 | 4.2 | 8 | 5.2 |
| 13 | 10 | 4.7 | 7 | 4.6 |
| 14 | 7 | 3.3 | 6 | 3.9 |
| 15 | 7 | 3.3 | 6 | 3.9 |
| 16 | 3 | 1.4 | 2 | 1.3 |
| 17 | 6 | 2.8 | 6 | 3.9 |
| 18 | 7 | 3.3 | 5 | 3.3 |
| 19 | 4 | 1.9 | 3 | 2.0 |
| 20 | 3 | 1.4 | 2 | 1.3 |
| 21 | 2 | 0.9 | 1 | 0.7 |
| 22 | 3 | 1.4 | 2 | 1.3 |
| 23 | 5 | 2.3 | 3 | 2.0 |
| 24 | 5 | 2.3 | 3 | 2.0 |
| 25 | 1 | 0.5 | 0 | 0.0 |
| 26 | 3 | 1.4 | 2 | 1.3 |
| 27 | 3 | 1.4 | 2 | 1.3 |
| 28 | 1 | 0.5 | 1 | 0.7 |
| 30 | 3 | 1.4 | 1 | 0.7 |
| 32 | 2 | 0.9 | 1 | 0.7 |
| 33 | 1 | 0.5 | 1 | 0.7 |
| 39 | 1 | 0.5 | 1 | 0.7 |
| 40 | 1 | 0.5 | 1 | 0.7 |
| 43 | 1 | 0.5 | 1 | 0.7 |
| 55 | 1 | 0.5 | 1 | 0.7 |
| 56 | 1 | 0.5 | 1 | 0.7 |
| 58 | 1 | 0.5 | 1 | 0.7 |
| 61 | 1 | 0.5 | 0 | 0.0 |
| 67 | 1 | 0.5 | 1 | 0.7 |
| 70 | 1 | 0.5 | 1 | 0.7 |

**Supplementary Table 1. Counts and percentages of samples collected at each timepoint (starting at day 0) for the samples sequenced using shallow shotgun method (columns 2, 3) and used in the shallow data profiling (after rarefaction, columns 4, 5).**

|  | **Controls** | |
| --- | --- | --- |
|  | **Before rarefaction** | **After rarefaction to 100, 000 features per sample** |
| Number of participants | 143 | 109 |
| Age, mean years | 52.6 | 52.7 |
| Sex |  | |
| Male (%) | 78 (54.5) | 53 (48.6) |
| Female (%) | 65 (45.5) | 56 (51.4) |
| Ethnicity |  | |
| White (%) | 143 (100.0) | 109 (100.0) |

**Supplementary Table 2. Demographics of the control group before and after rarefaction. The data includes the mean age, sex and ethnicity of the subjects.**
